# Normative data and standard operating procedures for static and dynamic retinal vessel analysis as biomarker for cardiovascular risk

**DOI:** 10.1038/s41598-021-93617-7

**Published:** 2021-07-08

**Authors:** Lukas Streese, Giulia Lona, Jonathan Wagner, Raphael Knaier, Andri Burri, Gilles Nève, Denis Infanger, Walthard Vilser, Arno Schmidt-Trucksäss, Henner Hanssen

**Affiliations:** 1 Department of Sport, Exercise and Health, Medical Faculty, University of Basel, Birsstrasse 320 B, 4052 Basel, Switzerland; 2 Institute of Biomedical Engineering and Informatics, Ilmenau University of Technology, Ilmenau, Germany

**Keywords:** Physiology, Ageing

## Abstract

Retinal vessel phenotype is predictive for cardiovascular outcome. This cross-sectional population-based study aimed to quantify normative data and standard operating procedures for static and dynamic retinal vessel analysis. We analysed central retinal arteriolar (CRAE) and venular (CRVE) diameter equivalents, as well as retinal endothelial function, measured by flicker light‐induced maximal arteriolar (aFID) and venular (vFID) dilatation. Measurements were performed in 277 healthy individuals aged 20 to 82 years of the COmPLETE study. The mean range from the youngest compared to the oldest decade was 196 ± 13 to 166 ± 17 µm for CRAE, 220 ± 15 to 199 ± 16 µm for CRVE, 3.74 ± 2.17 to 3.79 ± 2.43% for aFID and 4.64 ± 1.85 to 3.86 ± 1.56% for vFID. Lower CRAE [estimate (95% CI): − 0.52 (− 0.61 to − 0.43)], CRVE [− 0.33 (− 0.43 to − 0.24)] and vFID [− 0.01 (− 0.26 to − 0.00)], but not aFID, were significantly associated with older age. Interestingly, higher blood pressure was associated with narrower CRAE [− 0.82 (− 1.00 to − 0.63)] but higher aFID [0.05 (0.03 to 0.07)]. Likewise, narrower CRAE were associated with a higher predicted aFID [− 0.02 (− 0.37 to − 0.01)]. We recommend use of defined standardized operating procedures and cardiovascular risk stratification based on normative data to allow for clinical implementation of retinal vessel analysis in a personalized medicine approach.

## Introduction

Microvascular dysfunction is a key part of the aetiology and development of cardiovascular (CV) and neurovascular diseases. Static (SVA) and dynamic retinal vessel analysis (DVA) are both non-invasive diagnostic tools to investigate microvascular ageing in the cerebrovascular microcirculation as window to the heart^1^. Narrowing of central retinal arteriolar (CRAE) and widening of central retinal venular (CRVE) diameter equivalents have been associated with incidence stroke^2^, coronary heart disease^3^ and higher CV mortality^4^ and have been shown to be predictive for long-term CV outcomes^5–7^. The prediction of stroke^6^ and atherosclerosis-related CV events^5^ has been shown to be ten to 20% more accurate when analysing retinal vessel diameters on top of classic CV risk stratification.

Dynamic retinal vessel analysis has the potential to directly and non-invasively investigate microvascular endothelial function by measuring flicker light-induced dilatation (FID) over time. Reduced FID has been associated with higher CV risk such as obesity^8^, hypertension^9^, hypercholesterolemia^10^, pre-diabetes or type 2 diabetes^11^. Nägele et al. showed reduced FID in CV risk patients with a further decline in heart failure patients^12^. Impaired FID has been shown to be predictive for non-fatal and fatal CV events in multi-morbid end-stage renal disease patients^13^ as well as long-term major adverse CV events and survival rates in CV risk patients^14^.

A recent position paper of the European Society of Cardiology highlighted the potential to use retinal vessel analysis for the non-invasive diagnosis of microvascular dysfunction in cardiovascular precision medicine, but criticized the lack of essential normative data and standard procedures^15^. This study, for the first time, aimed to report normative data of a healthy cohort as well as standard operating procedures for static and dynamic retinal vessel analysis as a basis for implementation in the clinical routine.

## Materials and methods

### Study design

This single centre cross-sectional population-based study is part of the COmPLETE study^16^, which aimed to generate health- and performance-related normative data from a healthy Swiss cohort. The COmPLETE study included extensive phenotyping including physical fitness and CV screening. A detailed study protocol has been published previously^16^. Briefly, randomly selected districts of Basel and Basel-Stadt in Switzerland received unaddressed letters with study information and an invitation to participate in the study. The first contact was via telephone to check for the inclusion and exclusion criteria described below. Eligibility was further assessed on the first appointment where anthropometric data, macrovascular health, cardiac imaging, blood sampling, physical activity and fitness were examined. Static and dynamic retinal vessel phenotyping were performed on a separate appointment during the subsequent four weeks. This study, registered on ClinicalTrials.gov: NCT03986892, was approved by the Ethics Committee of North-western and Central Switzerland (EKNZ 2017-01451) and was planned and conducted considering the principles stated in the Helsinki declaration^17^. All participants signed a written informed consent before the first measurement.

### Inclusion and exclusion criteria

Healthy non-smoking men and women between 20 and 100 years of age with a body mass index (BMI) < 30 kg/m^2^ were included. Exclusion criteria were any history of CV disease, any CV medication, high systolic (≥ 140 mmHg) or diastolic (≥ 90 mmHg) blood pressure, any chronic or inflammatory disease, any acute or chronic eye disease such as macular degeneration, cataract, diabetic retinopathy, glaucoma or any other ocular disease, high intraocular pressure (IOP) (≥ 20 mmHg), current or past smoking status, pregnant or breastfeeding women, drug or alcohol abuse, compromising orthopaedic problems, any form of dementia, inability to follow the study instructions (for example language problems or psychologic disorders), diseases regarded as contraindication for maximal exertion.

### Sample characteristics

Height, body mass, body mass index, waist- and hip circumference, body fat, muscle mass, peak oxygen consumption as well as blood sampling were measured and performed at the first appointment with standard procedures as described previously^16^. Systolic and diastolic blood pressure (BP) were measured at the second appointment after ten minutes of rest in a sitting position in a room with controlled temperature and dimmed light, directly before the static retinal vessel analysis. BP was measured twice at the right upper arm using an oscillometric device (Bosch + Sohn GmbH, Jungingen, Germany). IOP was measured with the ICare PRO (Tiolat Oy, Helsinki, Finland) rebound tonometer.

### Static retinal vessel analysis

**Figure 1:**
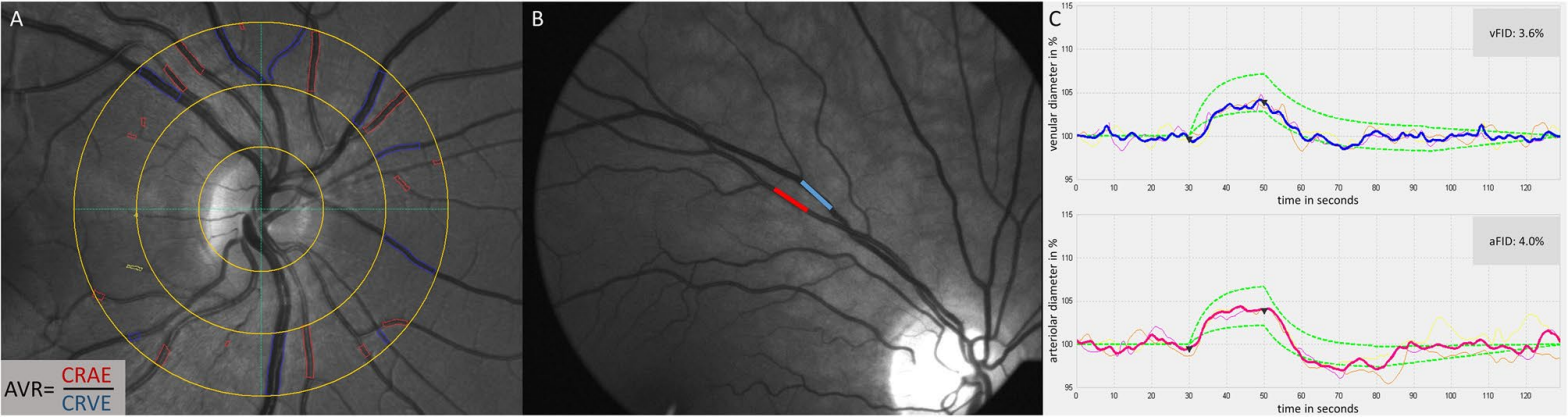
Static and dynamic retinal vessel analysis. (**A**) Static retinal vessel analysis with arteriolar (CRAE, red) and venular diameter equivalents (CRVE, blue). (**B**) Dynamic retinal vessel analysis (DVA) with one arteriolar (red) and one venular (blue) vessel segment. (**C**) Venular (blue) and arteriolar (red) response curves of DVA. Triangular marks indicating start and end of 20 s high frequency flicker light. *AVR* arteriolar-to-venular diameter ratio; *vFID* venular flicker-light induced maximal dilatation response; *aFID* arteriolar flicker light-induced maximal dilatation response.

Standard procedures to investigate SVA and DVA were performed using the Dynamic Vessel Analyzer (DVA®; IMEDOS Systems GmbH, Jena, Germany) based on a modified fundus camera (450FF; Carl Zeiss, Jena, Germany) after pupil dilatation of one eye using tropicamide 0.5%. Three images from one eye were taken with an angle of 50 degrees and the optic disc in the centre (Fig. 1A). Retinal arteriolar and venular diameter segments in the area of 0.5 to 1 disc diameter away from the optic disc margin were semi-automatically marked in all three images using a specified analyzing software (Vesselmap 2®; IMEDOS Systems GmbH, Jena, Germany) (Fig. 1A). Arteriolar and venular diameters from three images were averaged to CRAE and CRVE by use of the Parr–Hubbard formula, which has been described elsewhere^18^. The arteriolar-to-venular diameter ratio (AVR) was calculated from the CRAE and CRVE. Vessel diameters are presented in measuring units (mu). In the model of Gullstrand ‘s normal eye, 1mu relates to 1µm. Inter- and intra-observer interclass correlation coefficient for CRAE and CRVE ranged from 0.78 to 0.99^18,19^. We previously showed that the correlation coefficient for CRAE, CRVE and AVR ranged between 0.97 and 0.98 after re-analysis of 30 randomly selected images, proving a high reproducibility of this method to investigate retinal vessel diameters^20^. The investigator was blinded for participants’ sex and age.

### Dynamic retinal vessel analysis

**Figure 2:**
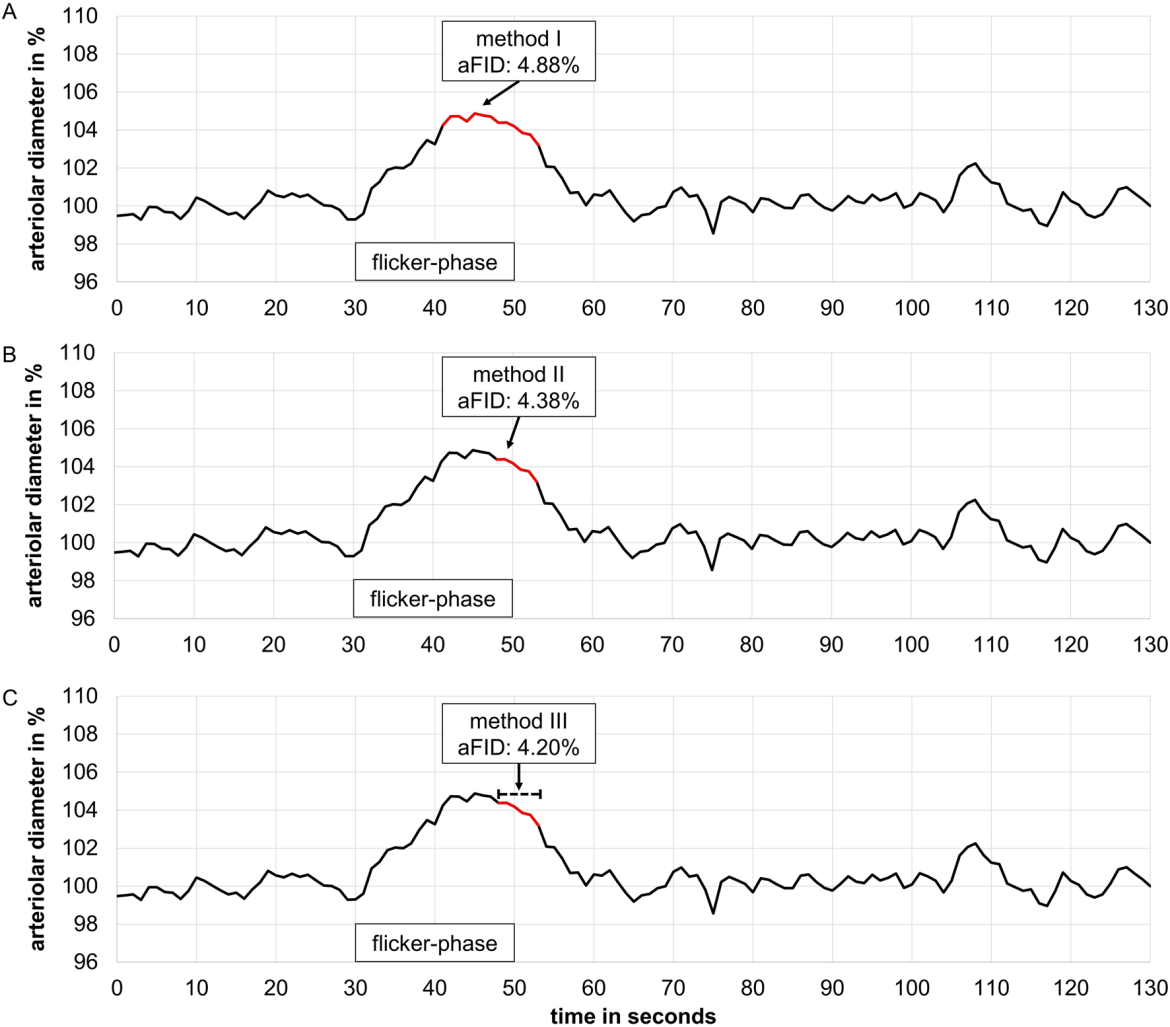
Method description to mark maximum flicker light-induced dilatation. (**A**) Method I marked the maximum flicker light-induced dilatation response (FID) ten seconds into the flicker till three seconds after the flicker phase (seconds 41–53, marked in red), based on the median curve of three identical flicker cycles. (**B**) Method II marked absolute maximum FID three seconds into the flicker till three seconds after the flicker phase (seconds 48–53, marked in red). (**C**) Method III averaged the last three seconds during and the first three seconds after the flicker phase (seconds 48–53, marked in red), which represents the standard report provided by the manufacturer (IMEDOS Systems). *aFID* arteriolar flicker light-induced dilatation response.

The DVA has been described in detail previously^16^. Briefly, one arteriolar and one venular segment, located in the upper temporal quadrant one to two optic disc diameters away from the optic disc edge, were marked (Fig. 1B) and their diameters were continuously measured over time (Fig. 1C). The standard procedure provided by IMEDOS Systems with three identical cycles with 50 s baseline, 20 s of high frequent flicker light (12.5 Herz) and 80 s recovery phase was applied. On the principles of neurovascular coupling^21^, this method measures retinal microvascular endothelial function non-invasively by analysing arteriolar (aFID) and venular flicker light-induced dilatation (vFID), as well as arteriolar constriction (aCON) using the integrated RVA software® (v.4.51; IMEDOS Systems GmbH, Jena, Germany). To date no gold standard to mark the maximum FID exists. Therefore, we evaluated the maximum FID, based on the median curve, in three different ways and provided percentile curves for every method. We marked maximum FID in the last ten seconds during, and three seconds after the flicker phase (seconds 41–53) in method I (Fig. 2A). Results of method I were included in the main manuscript. In method II, we marked the absolute maximum FID in the last three seconds during, and three seconds after the flicker phase (seconds 48–53, Fig. 2B), and in method III, we averaged the last three seconds during and the first three seconds after the flicker phase (Fig. 2C), which represents the standard report provided by the manufacturer (IMEDOS Systems). aCON was marked between the end of the flicker-phase and forty seconds after the flicker phase (seconds 50–90). The results of method II and III (Fig. 2B,C) were included in supplemental material. The correlation coefficient for aFID, vFID and aCON ranged between 0.98 and 0.99 after re-analysis of 30 random selected signals, providing a high reproducibility of method I to investigate retinal microvascular endothelial function. The day-to-day variability has been investigated previously and showed a high reproducibility with a correlation coefficient of 0.85 and a coefficient of variation of 5%^22^.

### Statistical analysis

Sample characteristics were descriptively presented by using mean and standard deviation, for the whole sample as well as for every age decade separately. Age- and sex-specific quantile curves were calculated using generalized additive models for location, scale, and shape (GAMLSS, R package version 5.1-7)^23^. The age-trajectories were modeled using P-splines. We used the Bayesian information criterion to select the conditional distribution that offered the best compromise between model complexity and goodness-of-fit. The model fits were inspected using diagnostic residual plots such as worm plots^24^ and Q–Q plots. Two linear regression analyses with CRAE and CRVE as dependent variable and age, sex, CRVE or CRAE, systolic and diastolic BP, BMI, IOP and peak oxygen consumption (VO2peak) as independent variables were performed to investigate associations of SVA parameters and a priori defined predictors. aFID, vFID or aCON as dependent variable and age, sex, AVR, vFID or aFID, systolic and diastolic BP, BMI, IOP and VO2peak as independent variables, were selected to investigate associations of DVA parameters and a priori defined predictors. Residual and Q-Q plots were used to test homoscedasticity and normal distribution that could be assumed for all models. R^2^ was partitioned using the calc.relimp function and the lmg method^25^. Pearson's product-moment correlations were calculated between retinal vessel parameters and age, parameters that reflect metabolic syndrome (waist circumference [WC], hemoglobin A1c [HbA1c], triglyceride and high-density lipoprotein [HDL]), as well as high-density C-reactive protein (hs-CRP) as an inflammatory marker. Linear regression models with heteroscedasticity-robust standard errors were conducted to investigate associations between CRAE, AVR and systolic as well as diastolic BP, between aFID, systolic and diastolic BP as well as between aFID and CRAE and AVR. The regression models were adjusted for age and sex and assumed a linear association between aFID and CRAE, AVR, systolic and diastolic BP. All statistical tests were performed two-sided with a significance level of 0.05. Statistics were performed and graphs were designed using R version 3.6.1 or later (R Foundation for Statistical Computing, Vienna, Austria).

## Results

We included 277 healthy participants between twenty and 82 years. Two participants were older than 80 years of age. These two participants were included in the 70 + age group. We documented 225 drop-outs after visit one (Supplement Fig. S1). Sample characteristics are described in Table 1. Sex distribution was nearly equal in all age decades, except of the two highest age groups with slightly more men than women. The overall CV risk was low due to the strict exclusion criteria. No statistically significant difference between men and women in the sample characteristics or retinal vessel phenotypes were observed.

**Table 1:** Sample characteristics.

	20–82 years n = 277 Mean ± SD	20–29 years n = 39 Mean ± SD	30–39 years n = 51 Mean ± SD	40–49 years n = 48 Mean ± SD	50–59 years n = 56 Mean ± SD	60–69 years n = 50 Mean ± SD	70 + years n = 33 Mean ± SD
Sex (f/m)	129/148	18/21	25/26	23/25	28/28	22/28	13/20
Age (years)	49 ± 16	25 ± 3	34 ± 3	45 ± 3	55 ± 3	64 ± 3	75 ± 4
Height (cm)	173 ± 9	174 ± 10	174 ± 8	174 ± 8	173 ± 9	172 ± 10	170 ± 9
Body mass (kg)	70 ± 11	71 ± 13	70 ± 11	71 ± 10	71 ± 11	71 ± 11	69 ± 10
BMI (kg/m^2^)	24 ± 3	23 ± 3	23 ± 3	23 ± 2	24 ± 2	24 ± 2	24 ± 3
WC (cm)	84 ± 9	81 ± 9	81 ± 9	82 ± 9	86 ± 8	88 ± 8	89 ± 10
HC (cm)	98 ± 6	98 ± 7	98 ± 7	98 ± 6	98 ± 5	97 ± 6	98 ± 6
Body fat (%)	22 ± 7	21 ± 8	20 ± 7	20 ± 7	24 ± 6	25 ± 8	26 ± 7
Muscle mass (kg)	31 ± 6	31 ± 7	31 ± 6	32 ± 6	30 ± 6	30 ± 6	28 ± 5
Systolic BP (mmHg)	124 ± 15	120 ± 16	121 ± 12	118 ± 14	123 ± 15	130 ± 15	133 ± 14
Diastolic BP (mmHg)	81 ± 8	78 ± 8	79 ± 7	80 ± 8	81 ± 9	83 ± 7	82 ± 10
HbA1c (%)	5.1 ± 0.3	5.0 ± 0.3	5.0 ± 0.3	5.1 ± 0.3	5.1 ± 0.4	5.3 ± 0.3	5.4 ± 0.3
Cholesterol (mg/dl)	220 ± 42	186 ± 41	201 ± 33	218 ± 45	238 ± 34	236 ± 38	237 ± 37
Triglyceride (mg/dl)	120 ± 69	123 ± 69	114 ± 73	112 ± 73	119 ± 55	134 ± 58	122 ± 92
HDL (mg/dl)	65 ± 16	61 ± 14	64 ± 16	68 ± 18	67 ± 16	64 ± 17	66 ± 15
LDL (mg/dl)	125 ± 29	101 ± 27	111 ± 22	123 ± 31	140 ± 24	138 ± 27	135 ± 24
Creatinine (mg/dl)	0.83 ± 0.14	0.81 ± 0.14	0.82 ± 0.14	0.85 ± 0.14	0.84 ± 0.15	0.81 ± 0.13	0.83 ± 0.17
Hs-CRP (mg/l)	1.3 ± 2.6	1.7 ± 3.7	1.1 ± 3.1	0.9 ± 1.5	1.0 ± 1.3	1.7 ± 2.7	2.1 ± 2.4
VO_2_peak (ml/min/kg)	38 ± 9	42 ± 9	42 ± 7	42 ± 9	36 ± 8	33 ± 8	28 ± 6
IOP (mmHg)	15 ± 3	15 ± 2	15 ± 2	14 ± 2	16 ± 4	16 ± 4	16 ± 4
CRAE (µm)	181 ± 16	196 ± 13	186 ± 15	181 ± 13	181 ± 13	174 ± 14	166 ± 17
CRVE (µm)	211 ± 16	220 ± 15	214 ± 13	211 ± 16	211 ± 14	207 ± 16	199 ± 16
AVR	0.86 ± 0.06	0.89 ± 0.06	0.87 ± 0.05	0.86 ± 0.06	0.86 ± 0.06	0.84 ± 0.05	0.83 ± 0.07
aFID (%)	3.65 ± 1.96	3.74 ± 2.17	3.66 ± 1.81	3.76 ± 2.10	3.04 ± 1.58	4.08 ± 1.77	3.79 ± 2.43
vFID (%)	4.23 ± 1.84	4.64 ± 1.85	4.49 ± 1.88	4.03 ± 2.06	4.19 ± 1.85	4.15 ± 1.75	3.86 ± 1.56
aCON (%)	− 1.65 ± 1.50	− 2.72 ± 1.97	− 1.99 ± 1.54	− 1.64 ± 1.27	− 1.29 ± 1.19	− 1.24 ± 1.27	− 1.11 ± 1.20

*SD* standard deviation; *BMI* body mass index; *WC* waist circumference; *HC* hip circumference; *BP* blood pressure; *HbA1c* hemoglobin A1c; *HDL* high-density lipoprotein; *LDL* low-density lipoprotein; *Hs-CRP* high-density C-reactive protein; *VO*_*2*_*peak* peak oxygen consumption; *IOP* intraocular pressure; *CRAE* central retinal arteriolar diameter equivalents; *CRVE* central retinal venular diameter equivalents; *AVR* arteriolar-to-venular diameter ratio; *aFID* arteriolar flicker light induced dilatation; *vFID* venular flicker light induced dilatation; *aCON* maximal arteriolar constriction.

### Static retinal vessel analysis

**Figure 3:**
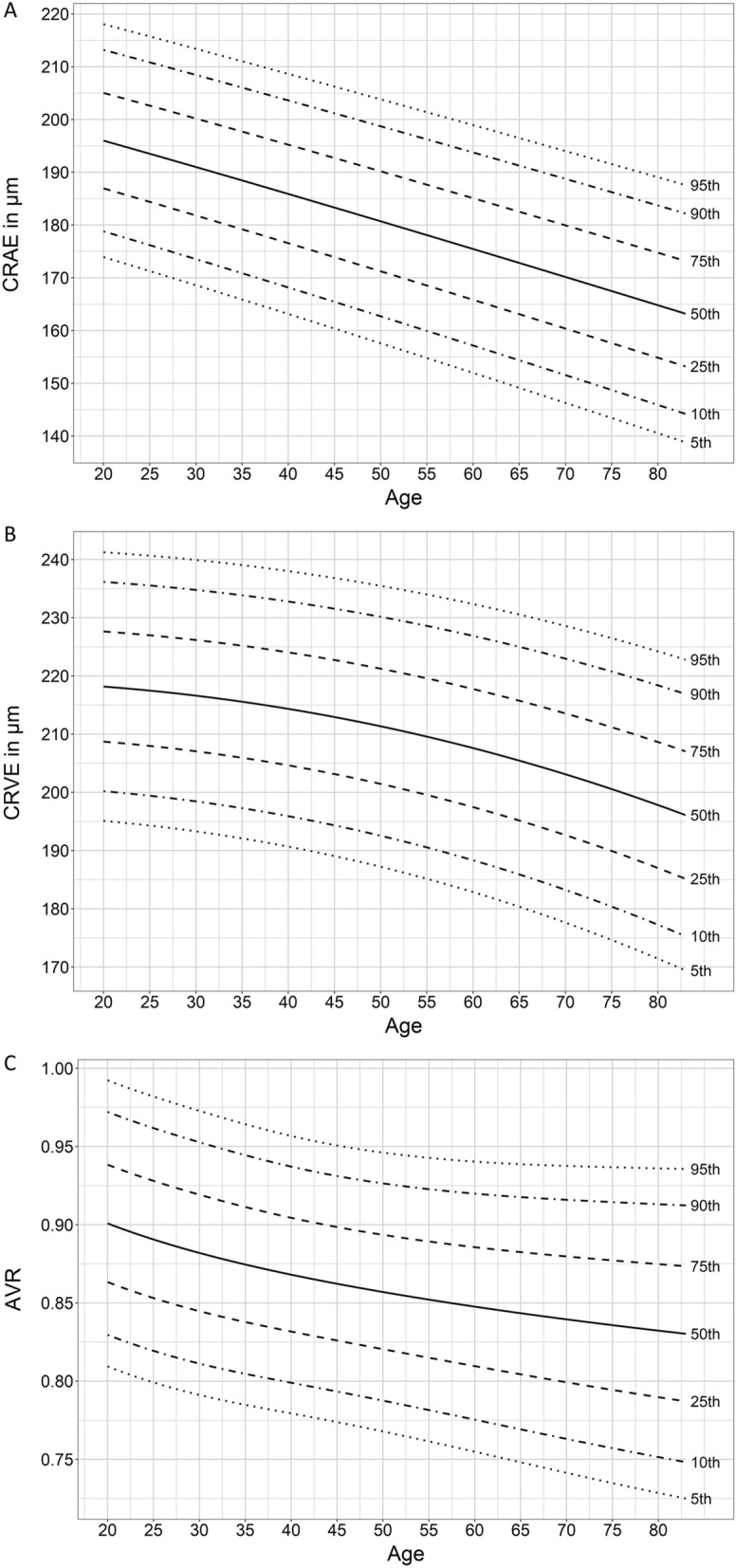
Normative data for static retinal vessel parameter. Quantile curves for (**A**) central retinal arteriolar diameter equivalents (CRAE), (**B**) central retinal venular diameter equivalents (CRVE) and (**C**) arteriolar-to-venular diameter ratio (AVR).

CRAE and CRVE were highest in the youngest age group with a continuous decline with increasing age. The average decline per decade was 6 µm for CRAE and 4 µm for CRVE. Figure 3 shows the percentile curves for CRAE, CRVE and AVR. Percentile curves for CRAE, CRVE and AVR separated for women and men are shown in Supplement Figs. S2 and S3. Strong evidence for negative associations [(estimate (95% confidence interval)] between CRAE [− 0.52 (− 0.61 to − 0.43), *p* < 0.001], CRVE [− 0.33 (− 0.43 to − 0.24), *p* < 0.001] as well as AVR [− 0.001 (− 0.001 to − 0.000), *p* < 0.001] and age were found. Additionally, wider CRAE were associated with wider CRVE [0.70 (0.62 to 0.78), *p* < 0.001]. Significant correlations were found between CRAE, CRVE as well as AVR and age as well as parameters reflecting the metabolic syndrome (Supplement Table S1).

All of the considered predictors together were able to explain 63% as well as 49% of the variation in CRAE and CRVE, respectively. The variation in CRAE was mainly explained by CRVE (33%), age (14%), systolic blood pressure (BP, 8%), diastolic BP (5%) and peak oxygen consumption (VO2peak, 3%). Further sample characteristics such as sex, BMI, and intraocular pressure (IOP) explained less than one percent of the variation in CRAE. Variation in CRVE was mainly explained by CRAE (40%), age (5%), diastolic BP (2%) and VO2peak (1%). Sex, systolic BP, BMI, and IOP explained less than one percent of variation in CRVE.

### Dynamic retinal vessel analysis

**Figure 4:**
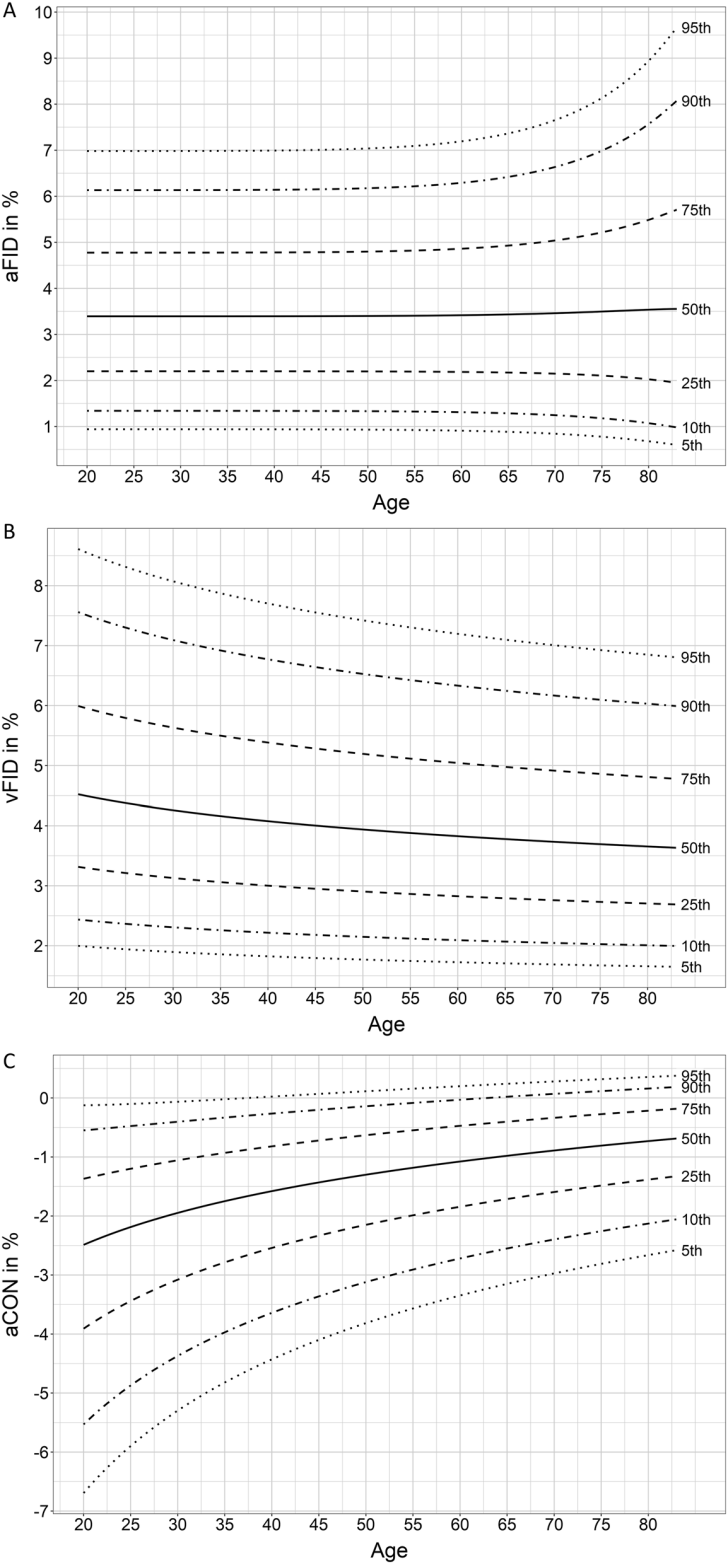
Normative data for dynamic retinal vessel parameter. Quantile curves for (**A**) arteriolar flicker-light induced maximal dilatation response (aFID), (**B**) venular flicker-light induced maximal dilatation response (vFID) and (**C**) maximal arteriolar constriction (aCON).

Percentile curves for aFID, vFID and aCON are described in Fig. 4. Separated percentile curves for women and men are shown in Supplement Figs. S4 and S5. Percentile curves for method II and III to quantify aFID and vFID are presented in Supplement Figs. S6–S9. Maximum FID marked by method I showed consistently higher aFID (mean aFID: 3.6%, 3.0% and 2.8%) and vFID (mean vFID: 4.2%, 3.9% and 3.6%) values compared to method II and III. No evidence for an association of aFID with age [0.00 (− 0.01 to 0.01), *p* = 0.852] was observed. vFID showed evidence for a negative association with age [− 0.01 (− 0.26 to − 0.00), *p* = 0.036]. A more pronounced aCON after the flicker phase was associated with younger age [0.03 (0.02 to 0.04), *p* < 0.001]. Significant correlations were found between aFID, vFID as well as aCON and sample characteristics (Supplement Table S1).

All of the considered predictors together were able to explain 23%, 19% and 22% of the variation in aFID, vFID as well as aCON. Variation of aFID was mainly explained by vFID (13%), systolic BP (4%) and diastolic BP (3%), as well as AVR (3%). Age, sex, BMI, IOP, and VO2peak explained less than one percent of variation in aFID. Variation of vFID was mainly explained by aFID (13%), sex (2%), AVR (1%), diastolic BP (1%) and age (1%). Systolic BP, BMI, IOP and VO2peak explained less than one percent of variation in vFID. Age (7%), aFID (5%), VO2peak (2%), sex (2%), AVR (1%), systolic BP (1%), and BMI (1%) mainly explained the variation in aCON.

### Blood pressure and combined static and dynamic retinal vessel analysis

**Figure 5:**
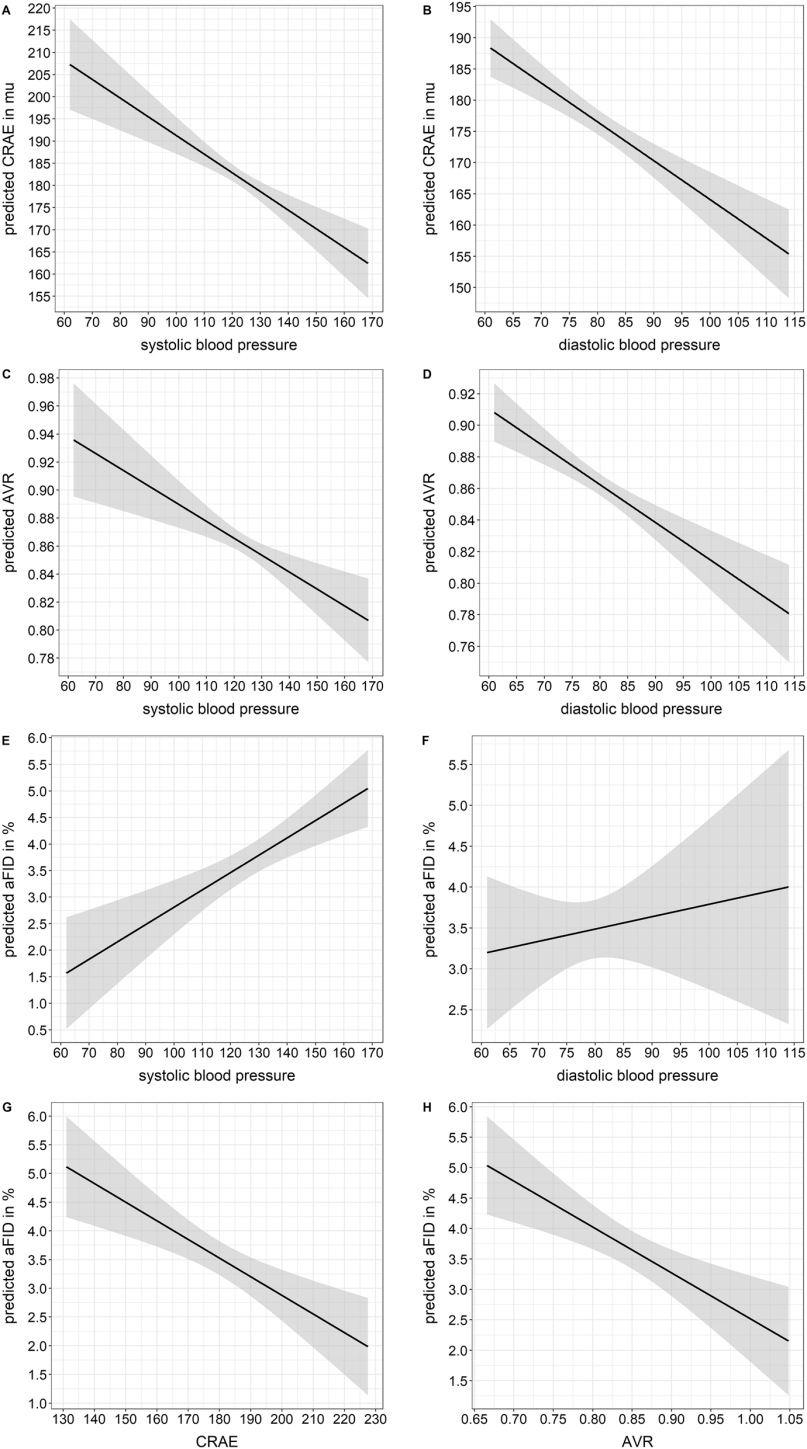
Associations of blood pressure, static and dynamic retinal vessel parameter. High systolic and diastolic blood pressure were associated with predicted lower CRAE (**A**,**B**), lower AVR (**C**,**D**) as well as higher aFID (**E**,**F**). Additionally, wider baseline diameters, reflected by wider CRAE or higher AVR, were associated with predicted lower aFID (**G**,**H**).* CRAE* central retinal arteriolar diameter equivalents; *AVR* arteriolar-to-venular diameter ratio; *aFID* arteriolar flicker-light induced maximal dilatation response.

Every ten mmHg systolic and diastolic BP increase resulted in narrower CRAE [systolic: − 2.79 (− 3.78 to − 1.81), *p* < 0.001; diastolic: − 6.22 (− 7.90 to − 4.55), *p* < 0.001] and lower AVR [systolic: − 0.001 (− 0.001 to − 0.001), *p* < 0.001; diastolic: − 0.021 (− 0.027 to − 0.014), *p* < 0.001] but a higher aFID [systolic: 0.33 (0.18 to 0.47), *p* < 0.001; diastolic: 0.53 (0.29 to 0.78), *p* < 0.001], corrected for age and sex (Fig. 5A–F). In addition, ten µm wider CRAE [− 0.32 (− 0.47 to − 0.18), *p* < 0.001] and 0.01 higher AVR [− 0.08 (− 0.11 to − 0.04), *p* < 0.001] resulted in lower aFID, corrected for age and sex (Fig. 5G,H).

## Discussion

This study provides normative data for static and dynamic retinal vessel analysis of a healthy population sample for the first time. Normative data of healthy cohorts are essential for researchers and clinicians to quantify retinal microvascular health and CV risk. We recommend use of a standard operating procedure (method I) for the assessment of maximum FID as compared to alternative methodological approaches. Our findings highlight the necessity to combine static and dynamic retinal vessel analyses in order to differentiate physiological variation from pathophysiology.

### Static retinal vessel analysis

Narrowing of CRAE, widening of CRVE, and a lower AVR have previously been associated with higher CV risk^5,7^. The constant narrowing of CRAE and the decrease in AVR across the age span seems to reflect normal vascular ageing, with an increasingly unfavorable microvascular phenotype at higher age. On a mechanistic level, we have previously shown that exercise and CV risk may determine retinal microvascular health by regulation of p66^Shc^ gene expression, a determinant of oxidative stress^20,26^. Interestingly, we found constant narrowing of CRVE with older age. The high correlation of CRAE and CRVE and the fact that the variation in CRVE was mainly explained by CRAE (40%), may lead to the assumption that venular narrowing across the age span may reflect physiological adaptations to CRAE narrowing. In healthy older individuals, relatively narrow CRVE are therefore representative of age-related remodeling rather than a putative reduction of CV risk. Higher age has previously been associated with CRAE narrowing^27,28^. The Gutenberg Health Study demonstrated a comparable linear decrease in CRAE, CRVE and AVR with increasing age in a healthy sub-cohort^28^. However, the authors only illustrated the lowest quartiles in their cohort (1^st^, 5^th^, and 10th quartiles), did not report retinal vessel diameters for each decade separately and calculated linear equations that presumed a linear relationship of vessel diameters and age. We decided to report the 5th, 10th, 25th, 50th, 75th, 90th, 95th quartiles, based on generalized additive models, to be able to interpret the vessel parameter distribution for the whole cohort. Based on these differences in data processing and presentation, the comparability with the Gutenberg Health Study is limited.

In our healthy cohort, in the absence of CV risk factors, retinal vessel diameters were significantly associated with determinants of metabolic syndrome. This strengthens the high sensitivity of retinal vessel diameters as a subclinical microvascular biomarker of CV risk. Especially BP seems to be a main regulator of retinal vessel diameters. This can be attributed to functional narrowing of retinal arterioles due to higher BP at the time of measurement, a physiological process of myogenic vasoconstriction in response to increased intraluminal pressure peaks, also known as the Bayliss effect. Persistence of high BP and subsequent CRAE narrowing may lead to structural remodeling and has been shown to be a subclinical marker for the development of hypertension^2,29^ and CV end-organ damage later in life^5,6^.

### Dynamic retinal vessel analysis

This study provides the first normative data for DVA. Healthy populations seem to show no relevant age-dependent decline of retinal arteriolar endothelial function. The highest aFID range was 3.04% to 4.08% between the age decades. Studies that used the same flicker protocol showed comparable results in healthy cohorts^12,30^. The lack of age-dependent reduction in arteriolar endothelial function was unexpected since, in general, higher age is associated with reduced endothelial function^31^. However, two studies have previously confirmed that aFID does not decrease with increasing age^32,33^.

The phenomenon of a seemingly invariant aFID in an ageing population needs to be verified. In order to do so it has to be highlighted that higher systolic and diastolic BP at the time of measurement were associated with higher aFID. Ten mmHg higher BP was associated with up to 0.53% higher aFID (Fig. 5E,F). In a previous study, we showed that BP-induced narrowing of arteriolar diameters at rest resulted in an increase in aFID^34^. This was likely explained by an increase in dilatation reserve after BP-induced constriction. It therefore seems to be essential to carefully interpret aFID under consideration of the actual BP at the time of measurement as well as the resting diameter of the arteriole, as further discussed below.

vFID was highest in the youngest age groups and showed a slight but constant decline with increasing age. However, the lowest mean vFID was 3.04 ± 1.58%, which was higher compared to CV risk patients, reported to have vFID values of about 1.1%^14^.

aCON was lowest in the youngest age group and increased with age, comparable to previous findings^32,33^. Age was the main predictor to explain the variation of aCON in our cohort. Our data showed a moderate to low correlation between aCON and age, waist circumference as well as HbA1c. Future studies will have to elucidate the clinical and added value of assessing aCON in addition to aFID and vFID.

### Combined static and dynamic retinal vessel analysis

Wider baseline diameters, reflected by wider CRAE and higher AVR, resulted in lower aFID (Fig. 5G,H). In comparison, previous studies in large arteries also showed that wider baseline diameters resulted in a reduced flow-mediated dilatation^35^. We previously showed that wider arteriolar baseline diameters resulted in a blunted FID, best explained by a reduced dilatation reserve in predilated arterioles. Potentially this may lead to a false diagnosis of microvascular endothelial dysfunction^30,34^. Based on these principles we would like to speculate that wider arteriolar vessel diameters at younger age may be associated with a reduced dilatation reserve and, subsequently, with a relatively low aFID in young and healthy individuals. As ageing is associated with higher BP and arteriolar narrowing this may well increase the dilatation reserve leading to a relative increase in aFID in healthy older individuals. Age-dependent narrowing of arteriolar baseline diameters therefore seem to be responsible for a “masked” age-related decline of retinal endothelial function. On the basis of our evidence-based assumption we strongly recommend to combine static and dynamic retinal vessel analysis to correctly verify retinal microvascular health in a personalized medicine approach.

### Standard procedures for static and dynamic retinal vessel analysis

With respect to standardizing general procedures, participants should refrain from any sports activity (sustained shortage of breath and sweating) 24 h before the examination, from alcohol intake 12 h prior to the examination and from eating at least two hours before the measurement. We recommend to measure BP directly before the retinal assessments after at least 10 min of rest in a sitting position in a temperature-controlled room. Three images of at least one eye should be taken to average CRAE, CRVE and AVR from these images. The biological variance of vasomotion is particularly high at younger age. The mean of three images reduces the biological variance of CRAE, CRVE and AVR and leads to more robust results. Standard static image analysis is to be performed by use of a semi-automated software device as described in the methods section and elsewhere^36,37^.

To date, no gold-standard method for the analysis of maximum FID exists. We would like to recommend use of a single valid standard procedure to establish comparability between cohort data and study results. This would allow for a more unified interpretation of retinal microvascular endothelial dysfunction in clinical routine, as recommended by the European Society of Cardiology^15^. With respect to the DVA protocol, the maximum FID was marked ten seconds into the flicker till three seconds after the flicker phase of the median curve (method I, seconds 41–53 in the standard protocol provided by the manufacturer). In principle, the initial dilatation response is mainly triggered by a repolarization of smooth muscle cells, followed by further dilatation induced by nitric oxide release^38,39^. Therefore, we avoided to mark the maximum FID during the first ten seconds of the flicker phase. The described method I has the advantage that examiners with little experience in interpreting retinal microvascular endothelial function can easily mark the maximum FID of the median curve in the last ten seconds during, and three seconds after the flicker phase (seconds 41–53). Additionally, we showed a high reproducibility with relatively higher dilatation response rates for method I as compared to method II and III. Method II and III only consider the last three seconds during and three seconds after the flicker phase, which can lead to an underestimation of maximum FID. Especially younger participants showed a very dynamic flicker response pattern. Averaging the last three seconds during and the first three seconds after the flicker phase (method III) or ignoring segments with potentially higher dilatation during the last ten seconds of flicker provocation by taking only one absolute value at the end of the flicker phase (method II), would lead to an incorrect and lower maximum FID (Supplement Figs. S6–S9).

Even though the provided software is intuitive to use, examiners need expert advice and training in order to handle the retinal fundus camera and to provide images and videos with a sufficient quality. Insufficient quality may affect the results and the evaluation of retinal microvascular health. We recommend certification of examiners by an experienced expert before data acquisition.

### Limitations

Our recommendations for clinical application are to combine static and dynamic retinal vessel analysis to correctly verify retinal microvascular health in a personalized medicine approach. Future research will have to deliver the evidence for a step by step approach for clinical decision making based on the combination of static and dynamic retinal vessel imaging data. Several research gaps remain and follow-up studies are mandatory to evaluate the beneficial effect of combining SVA and DVA for individual CV risk stratification and risk prediction. Previous studies showed ethnic differences for retinal vessel diameters^40^. Therefore, the unlimited transfer of these normative data to other ethnic groups is not possible. We need to report a potential selection bias, even if representative households were contacted. It is possible that individuals with a high interest in a health screening decided to participate.

## Conclusion

Retinal vessel analysis is a non-invasive, reproducible, and easily applicable diagnostic tool which offers a microvascular window to the heart. Our findings allow for a better understanding of retinal vessel physiology and differentiation of pathophysiology. The presented normative data are milestones towards clinical implementation of static and dynamic retinal vessel imaging in daily clinical routine. We recommend and define use of standardized operating procedures and normative values to pave the way for improvement of clinical decision making and CV risk stratification in a personalized medicine approach.

## Supplementary Information

Supplementary Information.

## Data Availability

The datasets generated during the current study are available from the corresponding author on reasonable request.
