# Supplementary material for: Normative data and standard operating procedures for static and dynamic retinal vessel analysis as biomarker for cardiovascular risk
Source: Sci Rep. 2021 Jul 8;11:14136. doi: 10.1038/s41598-021-93617-7 (PMC8266855; doi:10.1038/s41598-021-93617-7)
Supplement: Supplementary file 1 — Supplementary Information. [file 41598_2021_93617_MOESM1_ESM.pdf]

SUPPLEMENTAL MATERIAL

Normative data and standard operating procedures for static and dynamic retinal vessel analysis as biomarker for cardiovascular risk

Lukas Streese[1] PhD, Giulia Lona[1] PhD, Jonathan Wagner[1] PhD, Raphael Knaier[1] PhD, Andri Burri[1] M.Sc, Gilles Nève[1] M.Sc, Denis Infanger[1] PhD, Walthard Vilser[2] PhD, Arno Schmidt-Trucksäss[1] MD, Henner Hanssen[1]* MD

[1]Department of Sport, Exercise and Health, Medical Faculty, University of Basel, Basel, Switzerland

[2]Institute of Biomedical Engineering and Informatics, Ilmenau University of Technology, Ilmenau, Germany

Address for Correspondence

Prof. Henner Hanssen, FESC

Professor of Preventive Sports Medicine

Head of Preventive Sports Medicine & Systems Physiology

Department of Sport, Exercise and Health, University of Basel

Birsstrasse 320 B , 4052 Basel

Fax 041 (0)61 207 4748

email: henner.hanssen@unibas.ch

Table S1. Correlation coefficient and 95% confidence interval of correlations between static and dynamic retinal vessel biomarkers and cardiometabolic parameters in the healthy cohort.

	CRAE (μm)	CRVE (μm)	AVR	aFID (%)	vFID (%)	aCON (%)
Age (years)	**-0.51** (-0.59 to -0.42), *p* <0.001	**-0.34** (-0.44 to -0.23), *p* <0.001	**-0.30** (-0.41 to -0.19), *p* <0.001	0.01 (-0.11 to 0.13), *p*=0.852	**-0.13** (-0.24 to -0.01), *p*=0.036	**0.33** (0.22 to 0.44), *p* <0.001
WC (cm)	**-0.23** (-0.34 to -0.11), *p* <0.001	-0.04 (-0.16 to 0.08), *p*=0.535	**-0.27** (-0.38 to -0.15), *p* <0.001	-0.03 (-0.15 to 0.09), *p*=0.602	-0.09 (-0.21 to 0.03), *p*=0.139	**0.27** (0.15 to 0.37), *p* <0.001
BMI (kg/m^2)	**-0.15** (-0.27 to -0.03), *p*=0.012	-0.03 (-0.15 to 0.09), *p*=0.645	**-0.18** (-0.29 to -0.06), *p*=0.004	0.02 (-0.10 to 0.14), *p*=0.767	-0.05 (-0.17 to 0.07), *p*=0.455	**0.13** (0.01 to 0.25), *p*=0.036
Systolic BP (mmHg)	**-0.39** (-0.48 to -0.28), *p* <0.001	**-0.18** (-0.29 to -0.06), *p*=0.003	**-0.32** (-0.43 to -0.21), *p* <0.001	**0.20** (0.08 to 0.31), *p*=0.001	0.01 (-0.11 to 0.12), *p*=0.934	0.12 (-0.00 to 0.24), *p*=0.050
Diastolic BP (mmHg)	**-0.41** (-0.50 to -0.30), *p* <0.001	**-0.18** (-0.29 to -0.06), *p*=0.003	**-0.35** (-0.45 to -0.24), *p* <0.001	**0.21** (0.09 to 0.32), *p* <0.001	0.08 (-0.04 to 0.20), *p*=0.204	0.10 (-0.02 to 0.21), *p*=0.114

HbA1c (%)	**-0.13** (-0.25 to -0.01), *p*=0.031	**-0.13** (-0.25 to -0.01), *p*=0.032	-0.03 (-0.15 to 0.09), *p*=0.645	0.03 (-0.10 to 0.15), *p*=0.675	-0.04 (-0.16 to 0.08), *p*=0.550	**0.14** (0.02 to 0.26), *p*=0.020
Triglyceride (mg/dl)	-0.07 (-0.19 to 0.05), *p*=0.232	-0.07 (-0.19 to 0.05), *p*=0.247	-0.02 (-0.14 to 0.10), *p*=0.744	-0.04 (-0.16 to 0.09), *p*=0.554	-0.05 (-0.17 to 0.07), *p*=0.394	0.09 (-0.03 to 0.21), *p*=0.145
HDL (mg/dl)	0.04 (-0.08 to 0.16), *p*=0.484	-0.03 (-0.15 to 0.09), *p*=0.648	0.09 (-0.03 to 0.21), *p*=0.140	0.11 (-0.01 to 0.23), *p*=0.085	-0.04 (-0.16 to 0.08), *p*=0.500	0.03 (-0.09 to 0.15), *p*=0.603
Hs-CRP (mg/l)	-0.03 (-0.15 to 0.10), *p*=0.682	0.08 (-0.04 to 0.20), *p*=0.201	**-0.12** (-0.24 to -0.00), *p*=0.044	0.08 (-0.04 to 0.20), *p*=0.188	0.03 (-0.10 to 0.15), *p*=0.686	-0.08 (-0.20 to 0.05), *p*=0.225

Abbreviations: CRAE, central retinal arteriolar diameter equivalents; CRVE, central retinal venular diameter equivalents; AVR, arteriolar-to-venular diameter ratio; aFID, arteriolar flicker light induced dilatation; vFID, venular flicker light induced dilatation; aCON, maximal arteriolar constriction; WC, waist circumference; BMI, body mass index; BP, blood pressure, HbA1c, hemoglobin A1c; HDL, high-density lipoprotein; Hs-CRP, high-density C-reactive protein; significant correlations in bold.

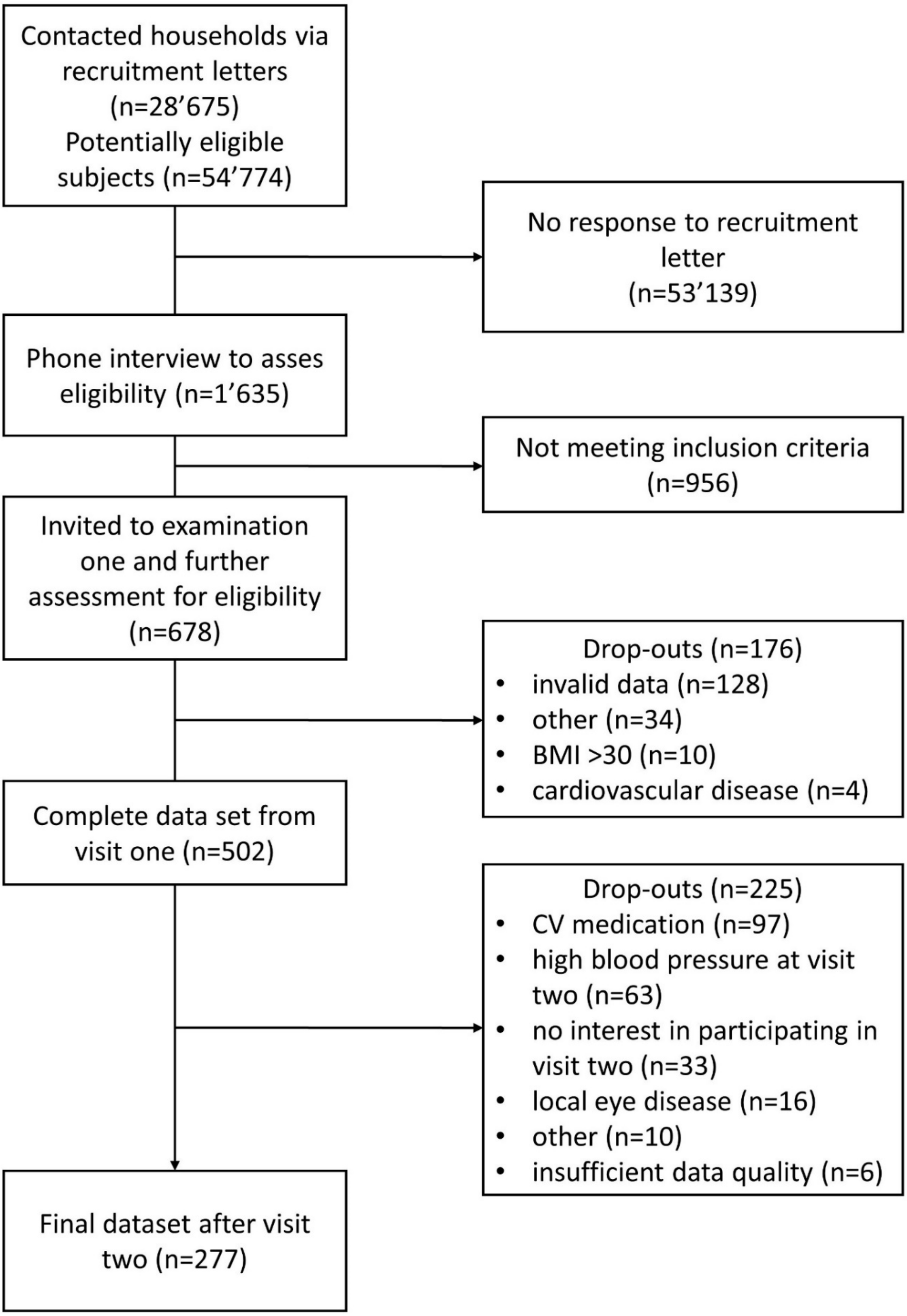

Figure S1. Flow-chart

BMI, body mass index; CV, cardiovascular.

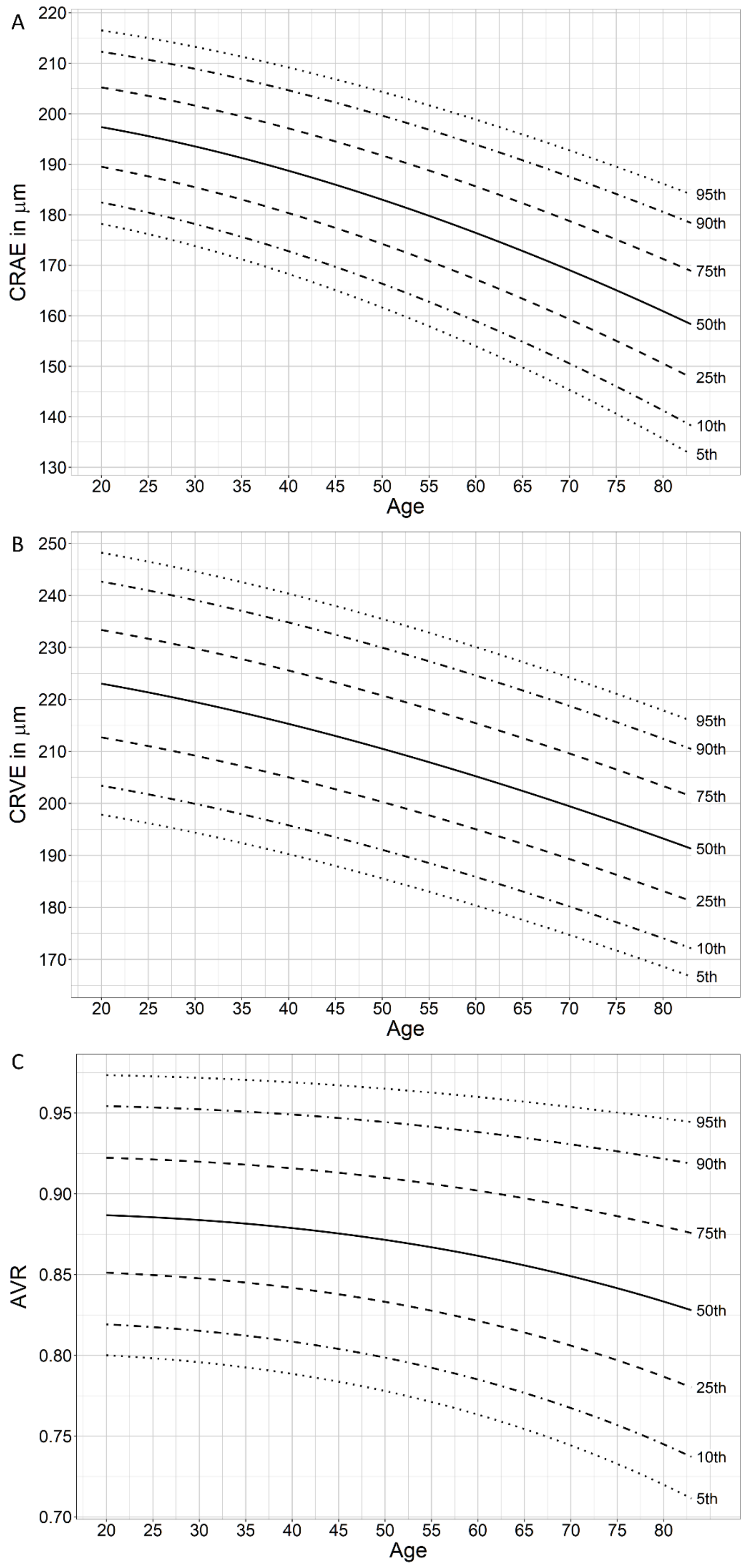

Figure S2. Normative data for static retinal vessel analysis in women: Quantile curves for (A) central retinal arteriolar diameter equivalents (CRAE), (B) central retinal venular diameter equivalents (CRVE) and (C) arteriolar-to-venular diameter ratio (AVR).

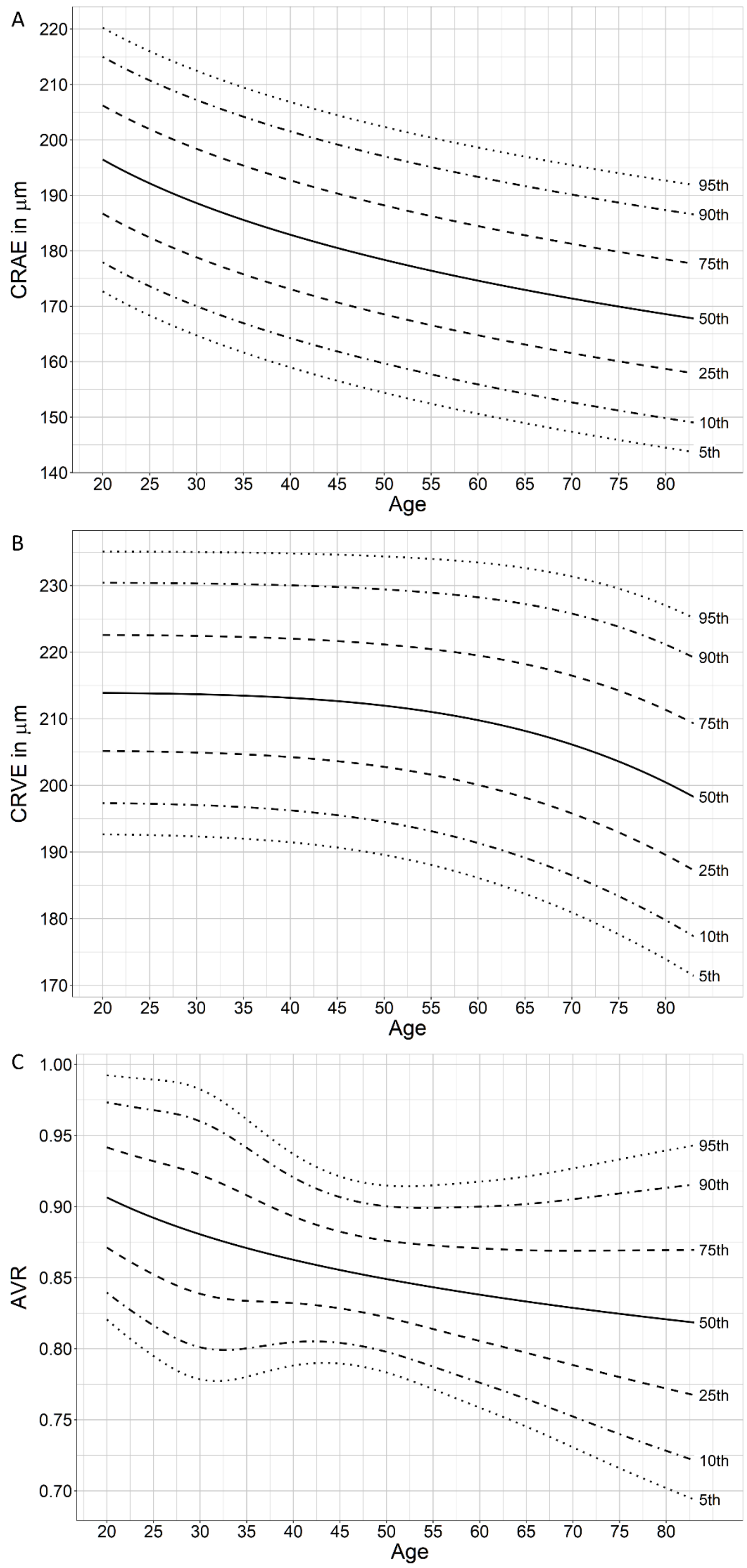

Figure S3. Normative data for static retinal vessel analysis in men: Quantile curves for (A) central retinal arteriolar diameter equivalents (CRAE), (B) central retinal venular diameter equivalents (CRVE) and (C) arteriolar-to-venular diameter ratio (AVR).

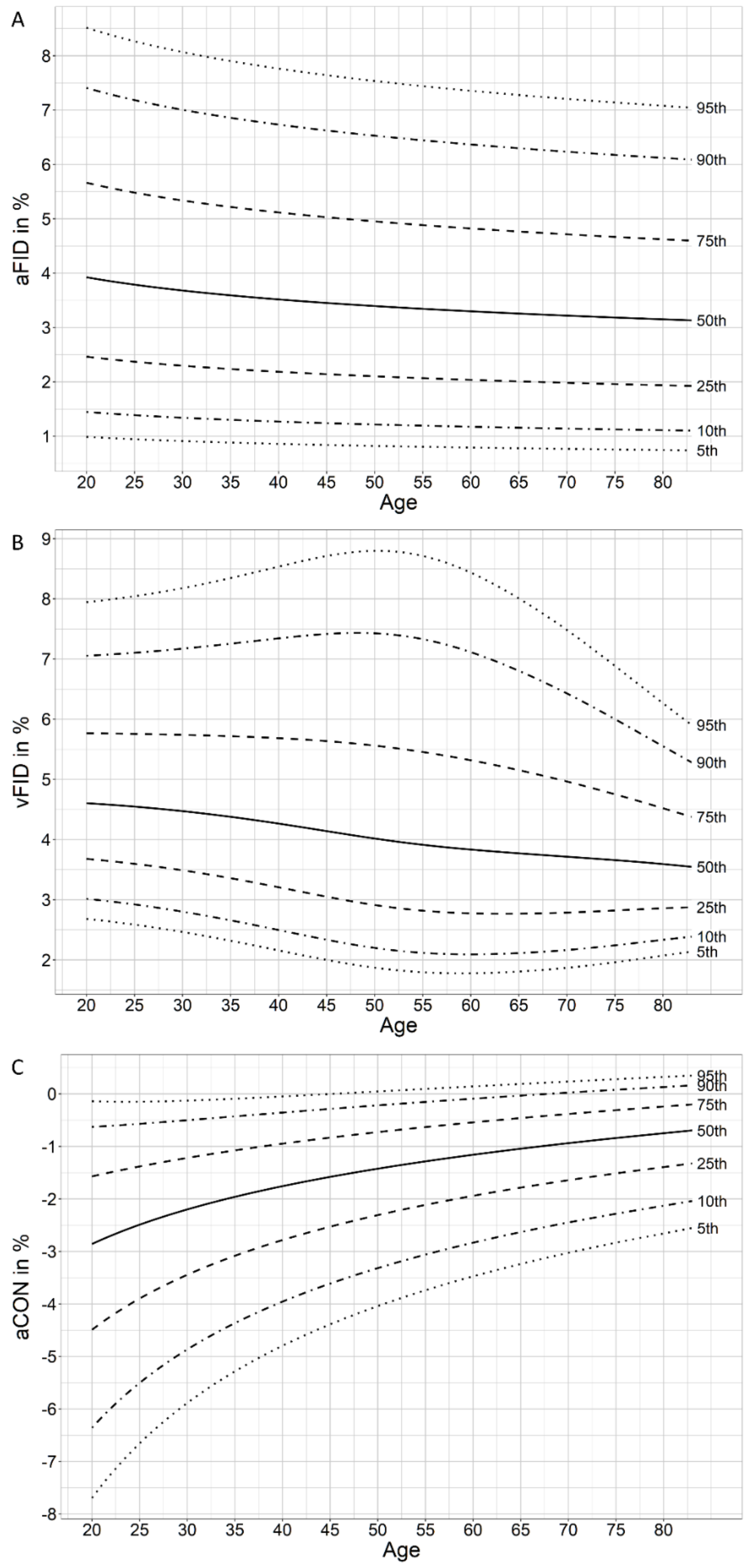

Figure S4. Normative data for dynamic retinal vessel analysis in women: Quantile curves for (A) arteriolar flicker light-induced maximal dilatation response (aFID), (B) venular flicker light-induced maximal dilatation response (vFID) and (C) maximal arteriolar constriction (aCON) of method I.

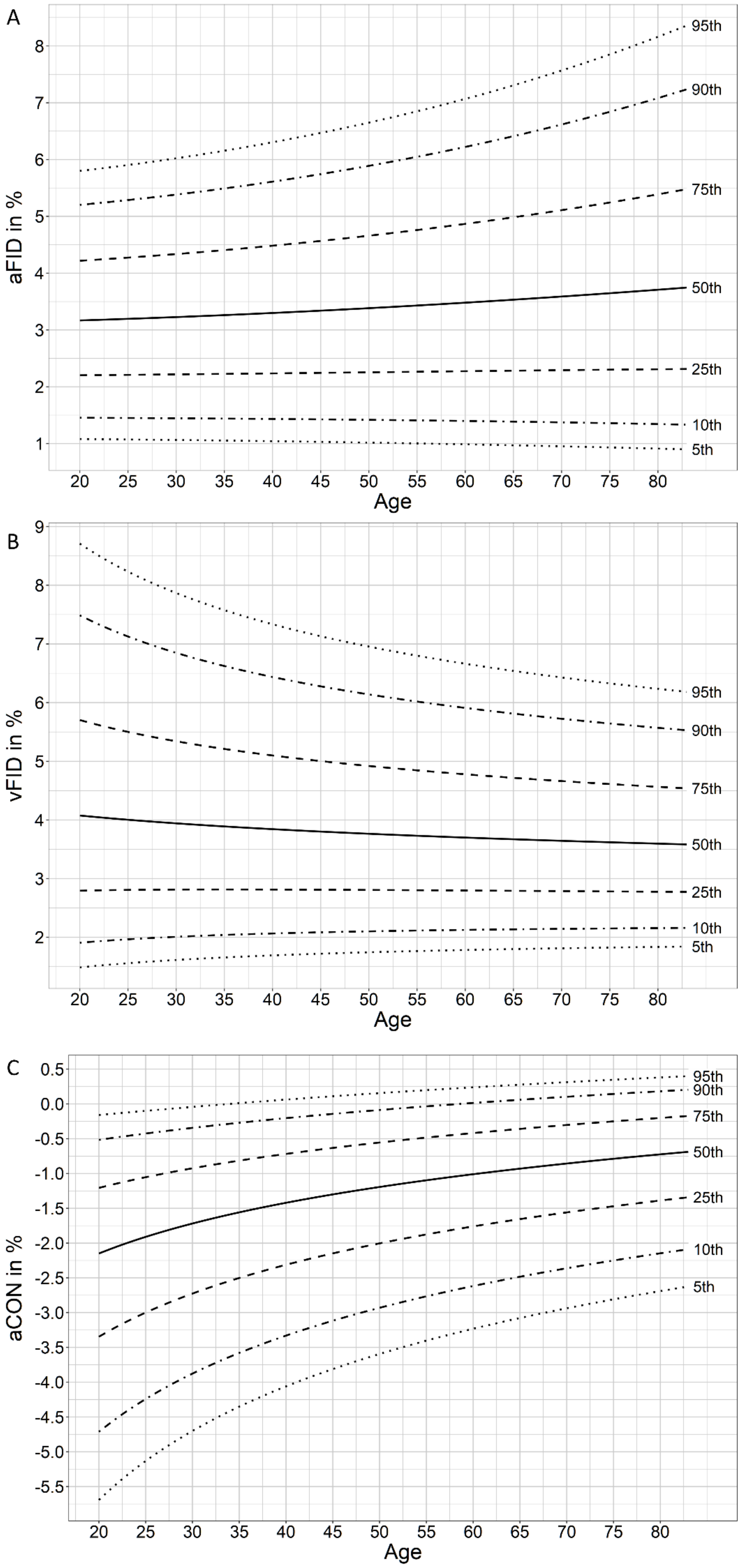

Figure S5. Normative data for dynamic retinal vessel analysis in men: Quantile curves for (A) arteriolar flicker light-induced maximal dilatation response (aFID), (B) venular flicker light-induced maximal dilatation response (vFID) and (C) maximal arteriolar constriction (aCON) of method I.

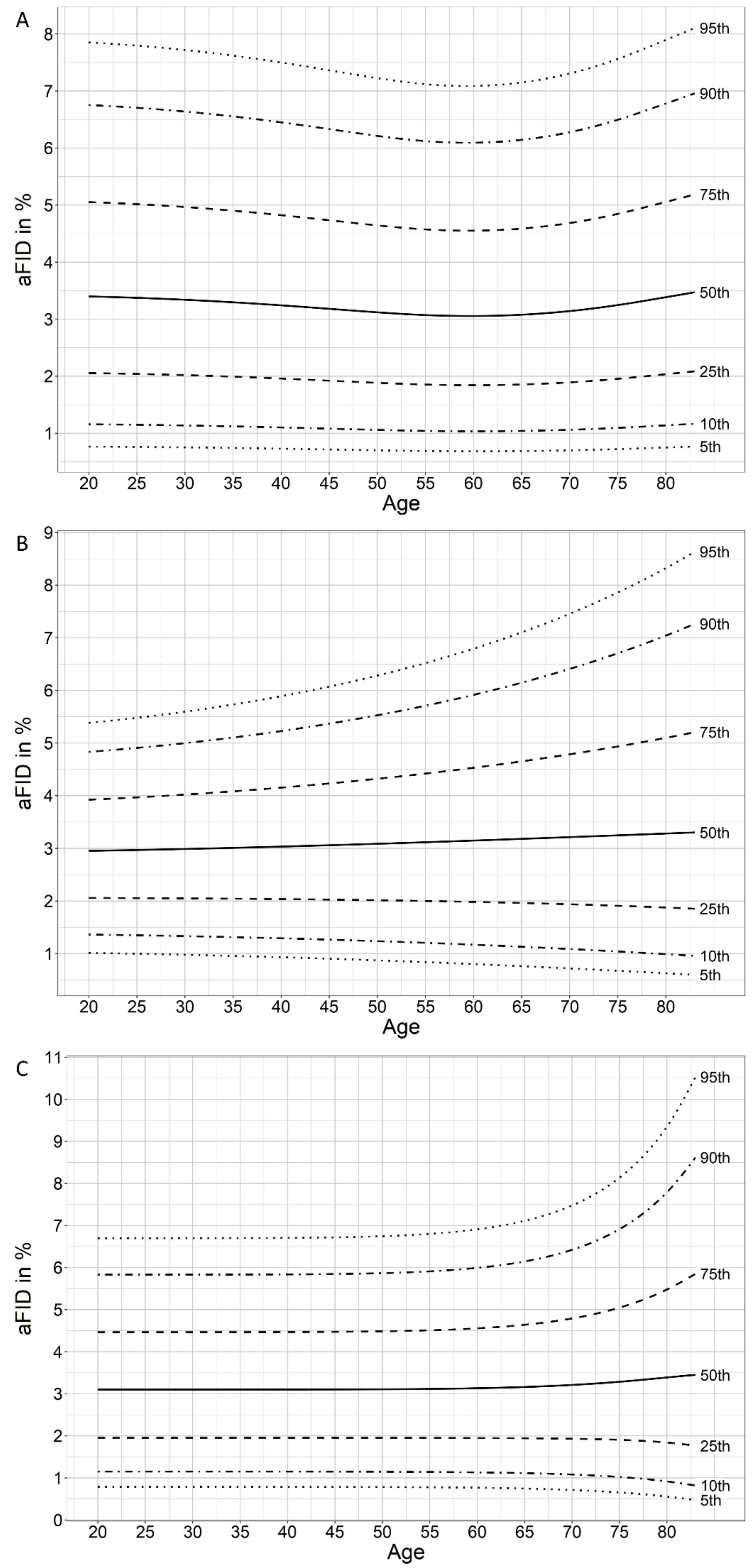

Figure S6. Normative data for arteriolar flicker light-induced maximal dilatation of method II: Quantile curves for (A) arteriolar flicker light-induced maximal dilatation (aFID) for women, (B) aFID for men and (C) combined aFID for women and men using method II.

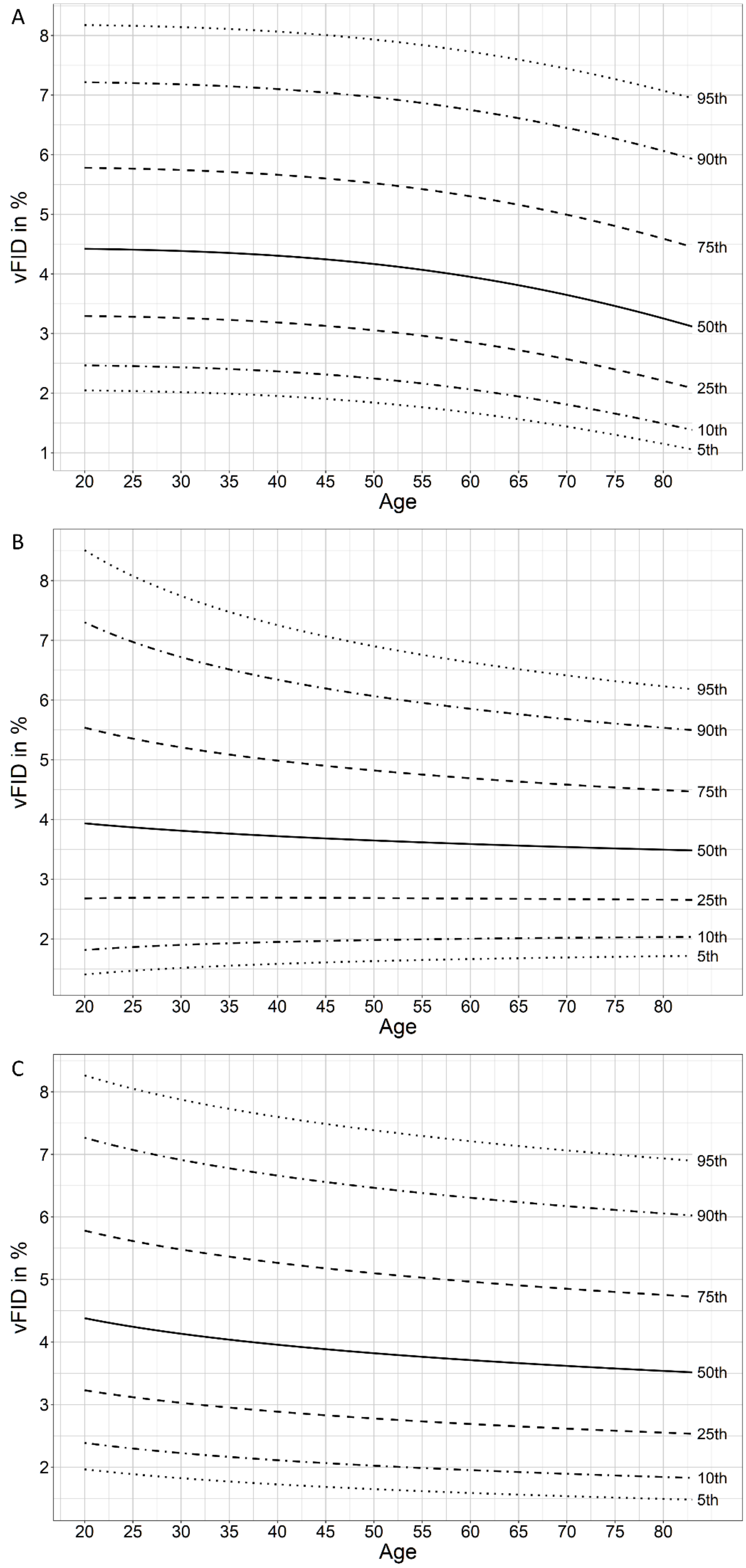

Figure S7. Normative data for venular flicker light-induced maximal dilatation of method II: Quantile curves for (A) venular flicker light-induced maximal dilatation (vFID) for women, (B) vFID for men and (C) combined vFID for women and men using method II.

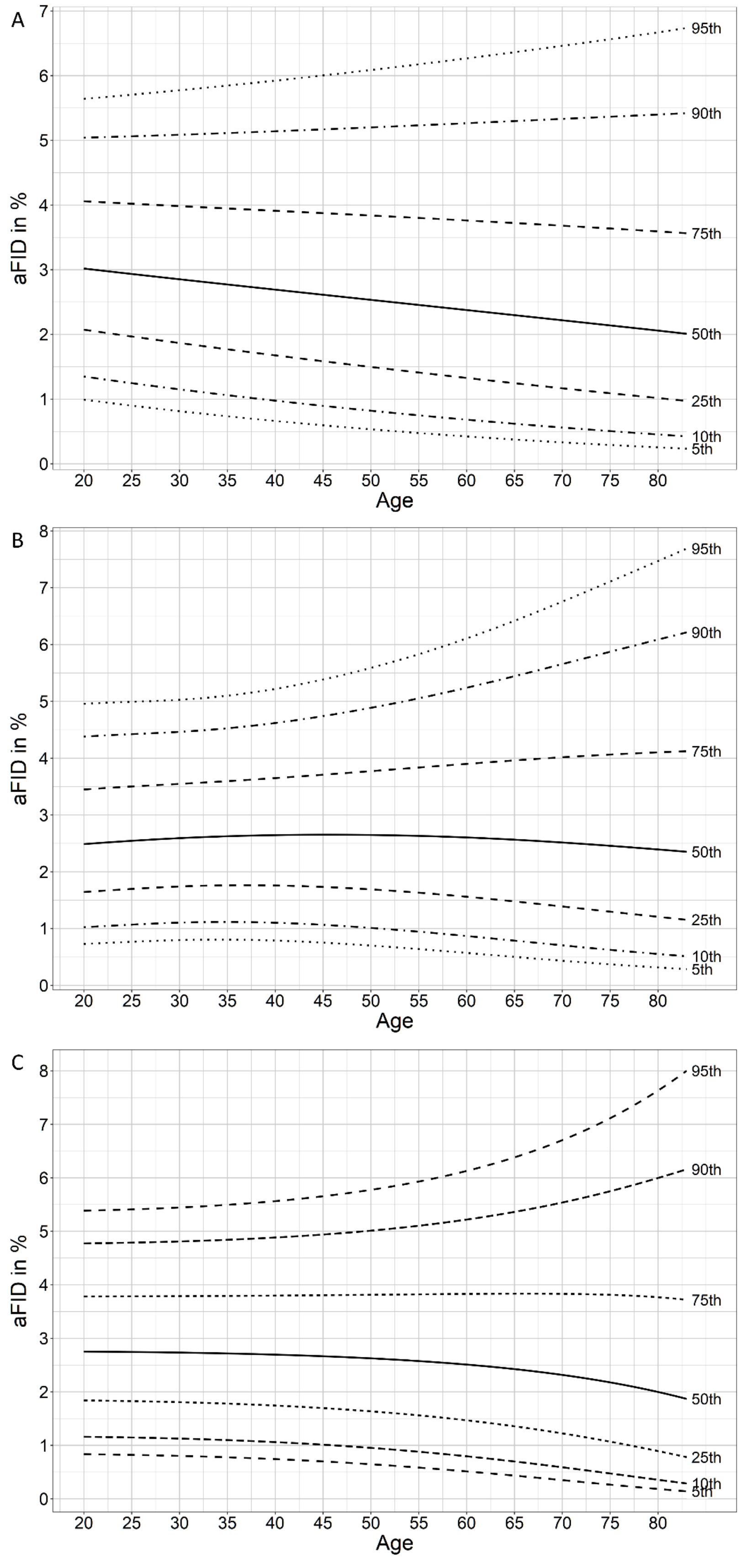

Figure S8. Normative data for arteriolar flicker light-induced maximal dilatation of method III: Quantile curves for (A) arteriolar flicker light-induced maximal dilatation response (aFID) for women, (B) aFID for men and (C) combined aFID for women and men using method III.

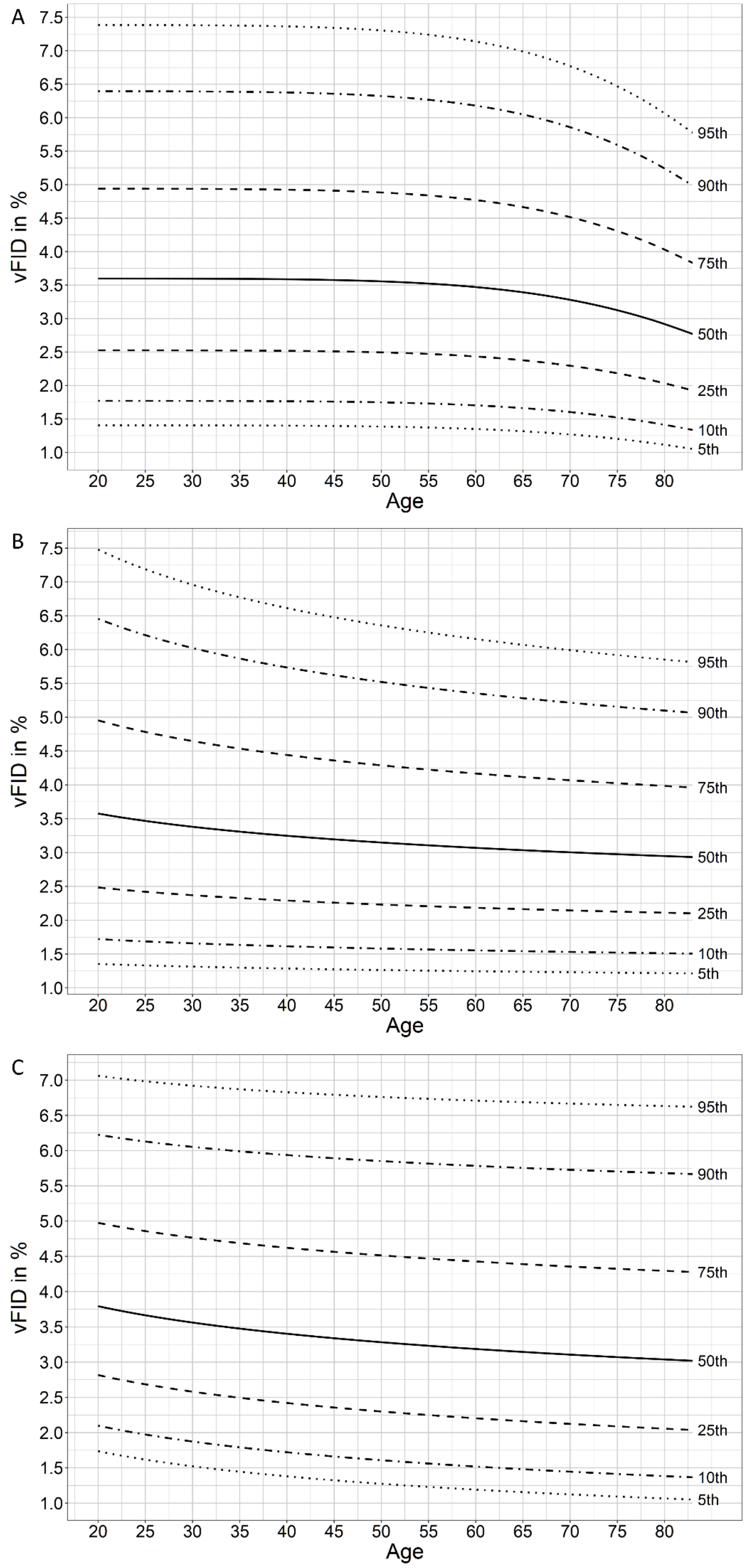

Figure S9. Normative data for venular flicker light-induced maximal dilatation of method III: Quantile curves for (A) venular flicker light-induced maximal dilatation (vFID) for women, (B) vFID for men and (C) combined vFID for women and men using method III.
